# Diet Quality among Cancer Survivors and Participants without Cancer: A Population-Based, Cross-Sectional Study in the Atlantic Partnership for Tomorrow’s Health Project

**DOI:** 10.3390/nu11123027

**Published:** 2019-12-11

**Authors:** Qianqian Gu, Trevor B. J. Dummer, John J. Spinelli, Rachel A. Murphy

**Affiliations:** 1 School of Population and Public Health, University of British Columbia, Vancouver, BC V6T 1Z3, Canada; christina.gu@alumni.ubc.ca (Q.G.); trevor.dummer@ubc.ca (T.B.J.D.); jspinelli@bccancer.bc.ca (J.J.S.); 2 Population Oncology, BC Cancer Agency, Vancouver, BC V5Z 1G1, Canada; 3 Cancer Control Research, BC Cancer Agency, 675 W 10th Ave, Vancouver, BC V5Z 1L3, Canada

**Keywords:** epidemiology, survivorship, healthy eating, dietary patterns, cancer

## Abstract

Cancer survivors are encouraged to have a healthy lifestyle to reduce health risks and improve survival. An understanding of health behaviors, such as diet, is also important for informing post-diagnosis support. We investigated the diet quality of cancer survivors relative to participants without cancer, overall and by cancer site and time from diagnosis. A cross-sectional study design within the Atlantic PATH study was used which included 19,973 participants aged 35 to 69 years from Atlantic Canada, of whom 1,930 were cancer survivors. A diet quality score was derived from a food frequency questionnaire. Comparisons of diet quality between cancer survivors and non-cancer controls, cancer site and years since diagnosis were examined in multivariable multi-level models. Cancer survivors had a mean diet quality of 39.1 out of 60 (SD: 8.82) and a higher diet quality than participants without cancer (mean difference: 0.45, 95% CI: 0.07, 0.84) after adjustment for confounders. Odds of high diet quality was greater in breast cancer survivors than participants without cancer (OR = 1.42, 95% CI: 1.06, 1.90), and higher among survivors diagnosed ≤2 years versus >10 years (OR = 1.71, 95% CI: 1.05, 2.80). No other differences by cancer site and years since diagnosis were observed. The difference in diet quality, although statistically significant, is unlikely to be meaningful, suggesting that cancer survivors have similar diet quality as participants without cancer. There was considerable room for dietary improvement regardless of cancer status, highlighting the need for dietary interventions, especially among cancer survivors, who are at higher risk for secondary health problems.

## 1. Introduction

Cancer survival has improved over the past decades due to early detection and advances in treatment [1]. It is estimated that 55% of people with cancer are alive 5 years after their diagnosis in Canada [1]. A cancer survivor is anyone who has ever had cancer since the time of diagnosis to the end of their life [1,2]. Cancer survivorship is accompanied by a higher risk of other chronic health conditions that may add financial, physical and mental health burden to cancer survivors, their families and the healthcare system [3]. The prevalence of comorbidities (e.g., diabetes, and congestive heart failure) among cancer survivors aged 65 years and older ranges from 31% for prostate cancer, 32% for breast cancer, 41% for colorectal cancer to 53% for lung cancer [4]. Comorbidities may also develop due to side effects of cancer treatments, or risk factors that are common with cancer, such as unhealthy diet, physical inactivity, smoking, and obesity [4,5,6,7]. 

Lifestyle changes including healthy eating are associated with reduced risk of comorbidities and improved survival after cancer diagnosis [8,9,10]. Health organizations such as the World Cancer Research Fund/American Institute for Cancer Research (WCRF/AICR) and the American Cancer Society (ACS) recommend cancer survivors consume a diet high in fruits and vegetables, legumes and whole grains, and low in red meat, processed meat, sugar, and fat, especially saturated fat [11,12,13]. 

Healthy eating is one of the most commonly reported positive health-related lifestyle changes after a cancer diagnosis [14]. Approximately 45% of adults with breast, prostate or colorectal cancer self-reported consuming more fruits and vegetables after diagnosis, and 26–28% self-reported consuming less red meat and fat [15]. However, despite motivation to improve diet, many survivors still fail to meet dietary guidelines. A study of 106 breast cancer patients reported that only 36–39% consumed more than five servings of fruits and vegetables, as recommended by the ACS guidelines [16]. Similar results were observed in an Australian study [17], in which the mean diet score of breast cancer patients was 33.2 out of 74, indicating poor adherence to the Australian dietary guidelines. 

There is mixed evidence about cancer survivors’ diet in comparison to non-cancer controls with most studies focusing on fruit and vegetable intake and selected nutrients [18,19,20,21,22,23]. A large study of 17,158 cancer survivors and 245,283 non-cancer controls reported that 73% of cancer survivors consumed less than five fruits and vegetables per day and had lower odds of meeting fruit and vegetable intake recommendations [20]. Similar findings were observed in a large study that examined fruit and vegetable intake of prostate cancer survivors [19]. A Japanese cohort study reported cancer survivors had higher intakes of fiber, potassium, calcium, B vitamins, vitamin C, zinc, copper, and manganese and a higher percentage of energy from protein but also had a higher percentage of energy from saturated fats and total fats [21]. Several other studies did not find any significant differences in dietary intakes of fruits and vegetables or macro- and micro-nutrients [17,24]. 

Only a few studies have compared overall diet quality of cancer survivors and participants without cancer (controls) [17,25]. A study of 1550 cancer survivors and 3100 controls reported a significantly lower Healthy Eating Index (HEI) 2010 score in cancer survivors although the difference was small (1.1/100 points) and became non-significant after adjustment for smoking status [25]. Our study aimed to understand whether cancer survivors engage in healthy eating and how this compares to those without cancer, by studying diet quality in a large cohort of Canadians. Additional aims were to examine how cancer survivors’ diet quality varies by cancer site and years since diagnosis.

## 2. Materials and Methods

### 2.1. Study Participants

Participants were drawn from the Atlantic Partnership for Tomorrow’s Health (PATH) study, part of the Canadian Partnership for Tomorrow Project (CPTP). The Atlantic PATH study recruited 34,169 people aged 30 to 74 years old between 2009 and 2015 from Prince Edward Island, New Brunswick, Nova Scotia, and Newfoundland and Labrador. Details of recruitment and study procedures have been published previously [26]. All participants provided written informed consent. All participants completed a core questionnaire that was common among CPTP regional cohorts, while 68% of participants completed a questionnaire that was unique to the Atlantic PATH study, including questions on dietary intake [27,28]. Physical measurements (e.g., height and weight) were assessed in a subset of participants. Data in the CPTP regional cohorts underwent a rigorous harmonization process to facilitate pan-Canadian comparisons. Data for this research was conducted on the first harmonized dataset which includes 31,173 participants aged 35–69. 

**Figure 1:**
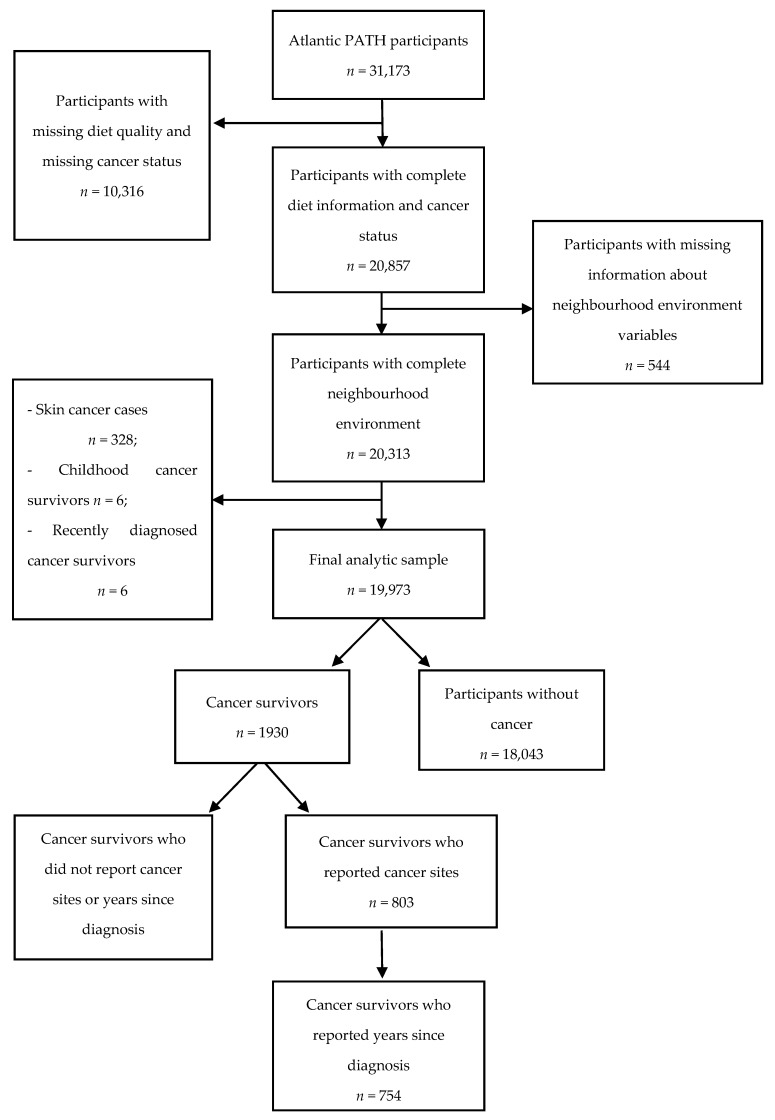
A flow chart for the selection of the final analytic sample.

Participants were excluded if there was missing data on diet quality or cancer status (*n* = 10,316, Figure 1), or if they had missing data for neighborhood environment (*n* = 544). All skin cancer cases were excluded as there was lack of information to distinguish cases of non-melanoma from melanoma (*n* = 328). Exclusions were also made for cancer diagnosis before the age of 15 (childhood cancer survivor, *n* = 6) and cancer diagnosis within the past 6 months of study enrollment (*n* = 6). As a result, 19,973 participants including 1930 cancer survivors and 18,043 participants without cancer were included in the analytical sample (Figure 1). About half of cancer survivors had missing information on site or years since diagnosis resulting in 803 cancer survivors for site-specific analyses and 754 cancer survivors for analyses based on years since diagnosis. 

### 2.2. Dietary Intake

Participants reported their usual daily dietary intake using a food frequency questionnaire (FFQ) that was unique to the Atlantic PATH study [27,28]. A total of 24 questions assessed the intake of fruit and vegetables, grains, dairy products, meat and alternatives, snacks, desserts, non-diet soft drinks, fats, sauces, and salt in the prior 12 months. Participants were asked to indicate the number of servings they consumed in a typical day of fruit and vegetables, grains, dairy and alternatives, and meat and alternatives. For other foods, participants were asked to indicate the usual frequency of consumption (times per day, times per week, times per month or rarely/never).

A diet quality score was specifically developed for the Atlantic PATH study using criteria adapted from the US and Canadian HEI (HEI-C) [29,30] as not all information was available to construct the HEI-C. Details of the Atlantic PATH diet quality score derivation have been previously published [27]. Briefly, five major criteria were used to assess participants’ intake of foods from the four adequacy food groups (i.e., fruit and vegetables, grains, dairy products, meat and alternatives) and a moderation group (i.e., snack, dessert and non-diet drink) on a scale of 0 to 10. Ten additional criteria were used to assess whether diets were meeting health-promoting recommendations: dark green vegetables, whole fruit and vegetables, whole grains, oils, low-fat dairy products, fish, meat alternatives, saturated fat, sauces, and salts. Component scores were summed for a diet quality score ranging from 0 to 60, with a higher score indicating better diet quality. The score was then categorized into quartiles, from which the highest quartile was considered to be high diet quality, whereas the lower three quartiles were considered low-to-intermediate diet quality.

### 2.3. Cancer Status

Participants who responded ‘yes’ to “has a doctor ever told you that you had cancer or a malignancy of any kind?” were categorized as cancer survivors. Participants were asked to indicate the cancer site(s) and age(s) at diagnosis(ses). When a person had multiple cancer diagnoses, information for the primary cancer was used in the present study. Years since diagnosis was calculated as the difference between age at Atlantic PATH baseline and age at diagnosis. 

### 2.4. Covariates

Covariates included sociodemographic factors (age, sex, ethnicity, education, marital status, and household income), lifestyle factors (smoking status, alcohol consumption, physical activity, and body mass index (BMI)), self-reported diagnosis of diabetes and myocardial infarction, urbanicity, province of residence, as well as area-level social and material deprivation and population density. Physical activity was evaluated using the short and long form International Physical Activity Questionnaire (IPAQ) [31]. The level of physical activity was categorized into low, moderate and high, according to the IPAQ scoring protocol [32]. Former smokers were those who smoked at least 100 cigarettes during their lifetime but did not smoke in the past 30 days. Current smokers were those who smoked at least 100 cigarettes in their lifetime and smoked in the past 30 days. All others were categorized as non-smokers. Participants were classified as abstainers (never consumed alcohol), former (consumed alcohol before but not over the past 12 months), occasional (≤2–3 times/month), regular (≥once/week but ≤2–3 times/week), and habitual drinkers (≥4–5 times/week). Urbanicity was recorded as urban or rural, as defined by the Postal Code Conversion File Plus (PCCF+), whereby urban areas were referred to as any census metropolitan areas or census agglomerations with a core population of at least 10,000 [33]. Material and social deprivation indices at the level of dissemination area were constructed using a principal component analysis using six neighborhood indicators from the census: the proportion of people without a high school diploma, the average individual income, the employment rate, the proportion of single-parent families, the proportion of people living alone, and the proportion of people who were separated, divorced or widowed [34,35,36]. Population density for each dissemination area (DA), a small geographic unit composed of one or more dissemination blocks, was calculated by dividing the population count by the land area, expressed in residents per km^2^. Material deprivation, social deprivation and population density were then categorized into tertiles separately. Neighborhood environment characteristics were linked to each individual by postal codes using the PCCF+ Version 6C [33]. 

### 2.5. Statistical Analysis

Multiple imputation was performed using the Multivariate Imputations by Chained Equation (MICE) package in R [37]. The missing values of education, household income, marital status, ethnicity, smoking status, alcohol consumption, physical activity, myocardial infarction, and diabetes were imputed. Age, sex, self-reported BMI, self-perceived health, and diet quality were included as auxiliary variables in the multiple imputation to make the MAR assumption more plausible and reduce bias [38]. A fully conditional specification method was adopted to impute different types of data using separate conditional distributions [37]. Forty imputed datasets were created to achieve sufficient statistical power [38] with 200 iterations to reach convergence [37]. 

Multi-level models, also known as mixed-effect models, were used as individuals are nested within forward sortation areas (FSAs). Although the neighborhood data were at the level of DAs, since the number of participants was too few within most DAs, FSAs were selected instead of DAs as the level of clusters in the multi-level models to achieve sufficient sample sizes. The non-independence of observations within the FSAs is accounted for in the mixed-effect models [39]. Differences in diet quality between cancer survivors and participants without cancer were considered with diet quality as a continuous score and dichotomized as high quality and low-to-intermediate diet quality. Linear mixed-effect models (LMMs) were used to obtain mean differences in diet quality, while generalized linear mixed-effect models (GLMMs) were used to derive odds ratios (ORs) of high diet quality. A random intercept was included in the models due to area-level variation in diet quality. For each multi-level model, backward elimination was used to remove potential confounders (age, sex, household income, education, marital status, ethnicity, smoking status, alcohol consumption, physical activity, BMI, diabetes, myocardial infarction, social deprivation, material deprivation, population density, urbanicity, and province of residence). At each step, the least significant variable was removed first until all variables in the model had a *p*-value < 0.20 [40]. As the covariates in each model vary, details on what was adjusted for are provided under the respective tables.

There were 22 cancer types reported among cancer survivors. However, data is presented only for the main cancer sites with sufficient sample size (*n* ≥ 30): breast, cervical, colorectal, prostate, thyroid, and uterine cancer. Diet quality was first compared among cancer survivors to determine whether differences existed and warranted further comparison of cancer sites with participants without cancer. Analyses were stratified by sex due to sex differences in diet quality, and the largest sample size was selected as the referent (prostate and breast cancer). Based on years since cancer diagnosis, four groups were created: ≤2 years, 2 to 5 years, 5 to 10 years and >10 years. The mean difference in diet quality and ORs of high diet quality were calculated by main cancer type and years since diagnosis in multivariable LMMs and GLMMs adjusting for confounders. 

The study was approved by the relevant Research Ethics Boards in each province. The protocol for the study of diet quality and cancer survivors was approved by the Research Ethics Board of the University of British Columbia (Certificate #H15-02854). All analyses were performed using R version 3.4.3 (R Foundation for Statistical Computing, Vienna, Austria).

## 3. Results

A total of 19,973 participants aged 35 to 69 years old were included, of whom 1930 were cancer survivors and 18,043 were participants without cancer. The mean diet quality (SD) of all participants was 38.8 (8.65) out of 60, corresponding to 65% adherence to Eating Well with Canada’s Food Guide. The diet quality component scores are presented in Table S1. On average, participants received high scores for meat and alternatives (mean: 8.95 out of 10), and low scores for grain products (mean: 4.32 out of 10) and snack/dessert/non-diet soft drinks (5.63 out of 10). Only 10.7% of participants consumed bread without added oil products, and 30.1% had at least one daily serving of meat alternatives such as bean, lentils and tofu.

Characteristics of participants in low-to-intermediate and high diet quality categories are shown in Table 1. Greater proportions of high diet quality were observed in female participants, participants 35 to 49 years old, participants with a household income of at least $75,000, those with a Bachelor’s degree or higher, non-smokers, occasional or regular alcohol drinkers, participants with high physical activity level, and with normal weight (*p* < 0.05). In addition, participants living in urban areas as well as those living in Nova Scotia and New Brunswick had better diet quality, which is indicated by the greater proportions of high diet quality. Additional comparison of characteristics between cancer survivors and participants without cancer are provided in Table S2. 

Results from LMMs of continuous diet quality are shown in Table 2. The mean diet quality of cancer survivors was 39.1 (SD: 8.82) which was 0.34 points higher (95% CI: −0.06, 0.75) than participants without cancer. The mean difference in diet quality score was 0.45 (95% CI: 0.07, 0.84) and became statistically significant after adjustment for potential confounders. Despite the statistical significance, the difference in the mean diet quality score was small given a total diet quality score of 60. This was reflected in the analysis of high-quality versus low-to-intermediate diet quality. A total of 26% cancer survivors had high diet quality, versus 25% of participants without cancer. The GLMMs also showed a non-significant unadjusted and adjusted odds ratio of high diet quality between cancer survivors and participants without cancer (Table 2). 

Among the 803 cancer survivors who provided information about their cancer sites, the mean diet quality for female breast cancer survivors (*n* = 226, Table 3) and female thyroid cancer survivors (*n* = 39) were 41.2 (SD = 7.49) and 42.0 (SD = 7.34), while the mean diet quality for cervical cancer survivors (*n* = 129) was 39.3 (SD = 8.57). Female colorectal cancer survivors (*n* = 39) and uterine cancer survivors (*n* = 34) had mean diet quality of 40.0 (SD = 7.42) and 41.0 (SD = 8.50), respectively (Table 3). Prostate cancer survivors (*n* = 65) had a mean diet quality of 36.2 (SD = 7.57, Table 3). There were no mean differences in diet quality by cancer type among male cancer survivors (data not shown). However, among women, cervical cancer survivors had a mean difference in diet quality score of -1.90 (95% CI −3.64, −0.15) compared to breast cancer survivors, and thus we proceeded with a comparison of diet quality between major cancer sites and participants without cancer. Compared to female participants without cancer, the mean diet quality was significantly higher in breast cancer survivors by 1.52 (95% CI: 0.47, 2.57) based on the adjusted LMM (Table 3). Similar results were observed in the adjusted GLMM; breast cancer survivors had 1.42 times (95% CI: 1.06, 1.90) higher odds of having a high diet quality compared to female participants without cancer. Participants of other cancer types including cervical, colorectal, thyroid, uterine, and prostate cancer had similar diet quality with their non-cancer counterparts of the same sex (Table 3).

Among the 754 cancer survivors who reported years since diagnosis, 37.9% were diagnosed more than 10 years prior (*n* = 286), 181 people were diagnosed 5 to 10 years prior, 166 were diagnosed 2 to 5 years prior, and 121 were diagnosed ≤2 years prior. There was no significant association between years since diagnosis and diet quality in the unadjusted LMM (Table 4). After adjusting for confounders, the mean difference in diet quality was 1.95 (95% CI: 0.32, 3.59) among cancer survivors diagnosed in the prior 6 months to 2 years compared to long-term survivors. Cancer survivors diagnosed between 2 to 5 years and 5 to 10 years had similar diet quality as long-term survivors. Similar results were observed in the adjusted GLMM; cancer survivors diagnosed in the prior 6 months to 2 years had 1.71 times (95% CI: 1.05, 2.80) higher odds of having a high diet quality compared to those diagnosed more than 10 years prior.

## 4. Discussion and Implications

This study is among the first to investigate Canadian cancer survivors’ overall diet quality and provides critical information on the prevalence of an important health behavior in cancer survivors in a large population-based cohort. Findings suggest that cancer survivors have a slightly better diet quality than participants without cancer when diet quality is considered as a continuous measure, although the difference of 0.45 units out of 60 is small and thus unlikely to have a health-related impact. When categorized into high and low-to-intermediate diet quality, the odds of having a high diet quality did not differ significantly between the two groups. We thus suggest that there is no meaningful difference in diet quality between cancer survivors and participants without cancer. However, diet quality differed by cancer site and years since diagnosis, with lower diet quality among cervical cancer survivors relative to breast, higher diet quality in breast cancer survivors compared to participants without cancer, and lower diet quality in long-term survivors. 

The finding of similar mean diet quality scores between cancer survivors and participants without cancer is in line with a US study by Zhang et al. that reported similar diet quality assessed via HEI scores after adjustment for smoking status [25]. An Australian study also found similar diet quality between breast cancer survivors and non-cancer controls [17]. While our study adds to the body of evidence on diet quality among cancer survivors compared to participants without cancer and provides a Canadian context, it does little to clarify whether differences in diet quality exist. It does, however, illustrate that many survivors and controls in this population do not meet national dietary guidelines for dietary intake and health-promoting diets. Of the individual components of the diet quality score, the prevalence of meeting guidelines for grain intake, snack/dessert/soft drinks, adding oil products to bread, and eating plant-based proteins and fish were the lowest, suggesting possible areas of focus for public health efforts and opportunities for cancer control intervention. 

Another area of potential focus of dietary efforts is long-term cancer survivors. The low diet quality observed among long-term cancer survivors compared to recently diagnosed patients fits with the literature that has shown the difficulty of long-term maintenance of healthy behaviors such as a higher prevalence of smoking in long-term cancer survivors [25] and weight gain and increased waist circumference in breast cancer survivors over time [41]. Conversely, cancer survivors who were diagnosed in the prior 2 years had the highest diet quality. This is similar to a study that showed cancer survivors within 2 years of diagnosis had a higher prevalence of consuming five or more fruit and vegetables than survivors diagnosed more than 2 years prior [42]. Conversely, other studies found no association between time since diagnosis and dietary intake including fruit and vegetables, whole grains, red meat, solid fats, added sugar, and alcohol [41,43]. The discrepancy may partly reflect the heterogeneity of study populations, for example, some focused on breast cancer survivors, while others included all cancer types, or all cancer types excluding those diagnosed <1 year prior. Cancer type is a likely confounder of these associations. Similar to other studies, we have shown that dietary intake may vary by cancer site [16,42,43], and survivorship is tightly linked to cancer type i.e., prostate cancer survivors are predominately long-term survivors, while lung cancer generally has shorter survival.

The present study showed that diet quality of breast cancer survivors was higher than that of participants without cancer. Our finding contradicts two studies from US and Australia that showed a similar diet between the two groups [16,44]. The discrepancy may reflect differences in methods, including minimal adjustment for confounders in prior studies and comparisons that were limited to individual food groups rather than an overall diet score. Although non-significant, the effect size for uterine and thyroid cancer was similar to breast. This suggests the non-significance reflects the smaller sample sizes and the higher diet quality among uterine and thyroid cancers may be meaningful. The finding of lower diet quality among cervical cancer survivors compared to participants without cancer, although non-significant, is notable. It is not immediately apparent why this cancer site may differ from the others investigated here, and further exploration is needed. The sample size did not permit exploration of variation in healthy eating by cancer sites other than prostate cancer in men, but the overall low mean diet quality among prostate cancer survivors is indicative of the need for additional support given the large number of prostate cancer survivors [45], high survival rates [45] and increased risk of nutrition-related chronic diseases such as diabetes and cardiovascular disease [46]. 

Strengths of the study include the large number of participants from diverse neighborhoods across all Atlantic provinces, which increases the generalizability of findings to this region. In addition, the large sample size enabled investigation of diet quality among all cancer types, by main cancer sites and years since diagnosis. The assessment of overall diet quality is also a strength. Fruits and vegetables are commonly used as an indicator of diet quality but are not as strongly linked to health outcomes as other diet components (e.g., processed meat) or overall diet. Other strengths of the study include the statistical models, which considered the potential geographical clustering of observations, as well as the simultaneous analysis of individual- and neighborhood-level variables. 

There are also limitations of this study. The cross-sectional study design makes it difficult to infer temporal relationships or causality. Secondly, the study is susceptible to selection bias. Participants were predominately female, which is typical of volunteer cohort studies; however, the predominately white ethnicity is reflective of the population in the Atlantic provinces. Most variables were self-reported, which can be subject to misclassification or measurement error. For example, almost half of the cancer survivors did not report cancer site or age at diagnosis. People also tend to over report their physical activity levels and intake of healthy foods and under report unhealthy foods due to social desirability [47]. However, the HEI-C and the diet quality score in the Atlantic PATH are based upon meeting serving size recommendations as well as nutritional adequacy and moderation components that are minimally affected by misreporting [29]. Diet quality in Atlantic PATH is not directly comparable to other diet quality indices such as the HEI-C or the US HEI due to limited dietary questions in the Atlantic PATH and differences in dietary guidelines between countries. The diet quality score in the Atlantic PATH has not been formally validated. However, the score performs well on three key indices of validity: content validity, construct validity and reliability. With respect to content validity, it includes key dietary recommendations of Canada’s Food Guide. The score can distinguish between groups with known differences in the quality of their diets, indicating construct validity. Better diet quality was observed in females, people with higher income and education, non-smokers, and physically active people in this study. A prior study reported inverse associations between adiposity and diet quality [27]. We also computed the standardized Cronbach’s alpha which was 0.74, indicating acceptable reliability. Lastly, diet quality was dichotomized into low-to-intermediate and high dietary quality which may have attenuated relationships. However, a sensitivity analysis comparing the lowest quartile of diet quality to the highest diet quality quartile in the GLMMs did not materially change results (data not shown) and results were similar to the analysis of diet quality as a continuous variable. 

In conclusion, this study provides insight into an important health behavior of cancer survivors in the Atlantic Provinces in Canada. Despite guidance promoting healthy lifestyle behaviors among survivors, findings suggest similar diet quality between survivors and non-cancer controls. The generally modest overall diet quality scores for both populations highlight the need for ongoing public health efforts to improve dietary intake. This may be particularly true among cancer survivors given that they are at a higher risk for chronic conditions and mortality [3,4], and thus the implications of a poorer diet may be particularly dire. 

## Figures and Tables

**Table 1:** Comparison of characteristics by categories of diet quality.

	Low-to-Intermediate Diet Quality	High Diet Quality	*p*-Value
	N = 14,965 N (%)	N = 5008 N (%)	
Cancer survivor			0.36
Yes	1429 (9.55)	501 (10.0)	
No	13,536 (90.5)	4507 (90.0)	
Age group, N (%)			<0.001
35–39	1440 (9.62)	567 (11.3)	
40–44	1698 (11.4)	671 (13.4)	
45–49	2199 (14.7)	857 (17.1)	
50–54	2692 (18.0)	857 (17.1)	
55–59	2788 (18.6)	820 (16.4)	
60–64	2534 (16.9)	788 (15.7)	
65–69	1614 (10.8)	448 (8.95)	
Sex			<0.001
Female	9705 (64.9)	4092 (81.7)	
Male	5260 (35.2)	916 (18.3)	
Marital status			0.97
Living without partners	2856 (19.1)	960 (19.2)	
Living with partners	12,077 (80.7)	4038 (80.6)	
Missing	32 (0.2)	10 (0.2)	
Ethnicity			0.08
White	13,006 (86.9)	4412 (88.1)	
Non-white	975 (6.52)	304 (6.07)	
Missing	984 (6.58)	292 (5.83)	
Household income			<0.001
0–24,999	679 (4.54)	173 (3.45)	
25,000–49,999	2483 (16.6)	730 (14.6)	
50,000–74,999	3057 (20.4)	916 (18.3)	
75,000–149,999	6109 (40.8)	2193 (43.8)	
>150,000	1784 (11.9)	711 (14.2)	
Missing	853 (5.70)	285 (5.69)	
Education			<0.001
≤high school	3140 (21.0)	696 (13.9)	
college	6182 (41.3)	1913 (38.2)	
≥Bachelor’s degree	5585 (37.3)	2390 (47.7)	
Missing	58 (0.39)	9 (0.18)	
Smoking status ^‡^			<0.001
Never	7319 (48.9)	2748 (54.9)	
Former	5894 (39.4)	1881 (37.6)	
Occasional	380 (2.54)	120 (2.40)	
Regular	1243 (8.31)	217 (4.33)	
Missing	129 (0.86)	42 (0.84)	
Alcohol consumption ^§^			<0.001
Abstainer	646 (4.32)	184 (3.67)	
Former drinker	1048 (7.00)	284 (5.67)	
Occasional drinker	6134 (41.0)	2145 (42.8)	
Regular drinker	4592 (30.7)	1662 (33.2)	
Habitual drinker	2456 (16.4)	714 (14.3)	
Missing	89 (0.59)	19 (0.38)	
Physical activity			<0.001
Low	3338 (22.3)	641 (12.8)	
Moderate	4482 (30.0)	1394 (27.8)	
High	6511 (43.5)	2785 (55.6)	
Missing	634 (4.24)	188 (3.75)	
BMI			<0.001
Normal	2825 (18.9)	1169 (23.3)	
Underweight	55 (0.37)	22 (0.44)	
Overweight	3725 (24.9)	1197 (23.9)	
Obese	2975 (19.9)	972 (19.4)	
Missing	5385 (36.0)	1648 (32.9)	
Diabetes			0.90
Yes	749 (5.01)	249 (4.97)	
No	14068 (94.0)	4713 (94.1)	
Missing	148 (0.99)	46 (0.92)	
Myocardial infarction			0.01
Yes	288 (1.92)	65 (1.30)	
No	14,577 (97.4)	4912 (98.1)	
Missing	100 (0.67)	31 (0.62)	
Urbanicity			0.03
Urban	10,689 (71.4)	3658 (73.0)	
Rural	4276 (28.6)	1350 (27.0)	
Province			<0.001
NL	1993 (13.3)	519 (10.4)	
PEI	290 (1.94)	87 (1.74)	
NS	9394 (62.8)	3177 (63.4)	
NB	3288 (22.0)	1225 (24.5)	
Social deprivation			0.14
Low	5407 (36.1)	1880 (37.5)	
Intermediate	4944 (33.0)	1646 (32.9)	
High	4614 (30.8)	1482 (29.6)	
Material deprivation			0.003
Low	6705 (44.8)	2371 (47.3)	
Intermediate	4793 (32.0)	1572 (31.4)	
High	3467 (23.2)	1065 (21.3)	
Population density			0.12
Low	4144 (27.7)	1375 (27.5)	
Intermediate	4912 (32.8)	1720 (34.4)	
High	5909 (39.5)	1913 (38.2)	

**^‡^** Non-smoker: has never smoked, former: has smoked at least 100 cigarettes but not within the past 30 days, occasional: smoked at least once within the past 30 days but not daily, regular: smoked daily. All other participants were categorized as non-smokers. **^§^** Abstainer: never consumes alcohol, former: has consumed alcohol before but not over the past 12 months, occasional: ≤2–3 drinks month over the past 12 months, regular: drinks ≥once/week but ≤2–3 times/week, habitual drinkers: drinks ≥4–5 times/week. NB; New Brunswick, NL; Newfoundland, NS; Nova Scotia, PEI; Prince Edward Island.

**Table 2:** Mean difference in diet quality scores (95% CI) and OR of high diet quality (95% CI) of cancer survivors compared to participants without cancer.

	N	Mean (SD)	Mean Difference (95% CI)		OR (95% CI)	
			Model 1	Model 2a	Model 1	Model 2b
Cancer status						
Non-cancer	18,043	38.9 (8.63)	reference	reference	reference	reference
Cancer survivors	1930	39.1 (8.82)	0.34 (−0.06, 0.75)	0.45 (0.07, 0.84) *	1.05 (0.95, 1.17)	1.08 (0.97, 1.21)

Model 1 is unadjusted. Model 2a is adjusted for age, sex, household income, highest education, marital status, ethnicity, smoking status, alcohol consumption, physical activity, diabetes, social deprivation, urbanicity, and province of residence. Model 2b is adjusted for age, sex, household income, highest education, smoking status, alcohol consumption, physical activity, diabetes, population density, and province of residence. * *p* < 0.05. Note that mean differences in Model 1 vary slightly from values obtained by calculating the difference between the reference group mean and subsequent cancer site means due to biases inherent in linear mixed-effect models.

**Table 3:** Mean difference in diet quality scores (95% CI) and OR of high diet quality (95% CI) with selected cancer sites compared to participants without cancer by sex.

	N	Mean (SD)	Mean Difference (95% CI)		OR (95% CI)	
			Model 1	Model 2a	Model 1	Model 2b
Females						
Cancer status/site						
Non-cancer	12,393	40.0 (8.26)	Reference	Reference	Reference	Reference
Breast	226	41.2 (7.49)	1.13 (0.04, 2.22) *	1.52 (0.47, 2.57) *	1.24 (0.94, 1.64)	1.42 (1.06, 1.90) *
Cervical	129	39.3 (8.57)	−0.62 (−2.05, 0.80)	−0.31 (−1.69, 1.07)	0.80 (0.54, 1.20)	0.86 (0.57, 1.30)
Colorectal	39	40.0 (7.42)	0.13 −2.45, 2.72)	0.84 (−1.65, 3.33)	0.84 (0.41, 1.74)	1.05 (0.50, 2.19)
Thyroid	39	42.0 (7.34)	1.93 (−0.66, 4.51)	1.87 (−0.62, 4.36)	1.65 (0.87, 3.13)	1.72 (0.89, 3.31)
Uterine	34	41.0 (8.50)	1.24 (−1.53, 4.01)	1.56 (−1.11, 4.24)	0.76 (0.34, 1.69)	0.85 (0.38, 1.92)
Males						
Non-cancer	5650	36.0 (8.79)	Reference	Reference	Reference	Reference
Prostate	65	36.2 (7.57)	0.17 (−1.97, 2.32)	0.38 (−1.72, 2.48)	0.80 (0.38, 1.69)	0.82 (0.39, 1.76)

Model 1 is unadjusted. Model 2a is adjusted for age, household income, highest education, marital status, smoking status, alcohol consumption, physical activity, diabetes, myocardial infarction, urbanicity, province of residence, and social deprivation. Model 2b is adjusted for age, household income, highest education, ethnicity, smoking status, physical activity, diabetes, myocardial infarction, and population density. * *p* < 0.05. Note that mean differences in Model 1 vary slightly from values obtained by calculating the difference between the reference group mean and subsequent cancer site means due to biases inherent in LMM.

**Table 4:** Mean difference in diet quality score among cancer survivors (N = 754) by years since cancer diagnosis, results from unadjusted and adjusted LMMs.

	N	Mean Diet Quality (SD)	Mean Difference (95% CI)		OR (95% CI)	
			Model 1	Model 2a	Model 1	Model 2b
Years since cancer diagnosis						
>10 years	286	39.4 (8.49)	Reference	Reference	Reference	Reference
>5 years ≤10 years	181	39.5 (7.83)	0.04 (−1.50, 1.57)	0.34 (−1.08, 1.76)	0.99 (0.64, 1.53)	1.11 (0.71, 1.73)
>2 years ≤5 years	166	39.5 (8.24)	0.06 (−1.52, 1.64)	0.83 (−0.62, 2.28)	1.26 (0.82, 1.93)	1.45 (0.94, 2.26)
≤2 years	121	39.9 (8.34)	0.43 (−1.32, 2.18)	1.95 (0.32, 3.59) *	1.36 (0.85, 2.18)	1.71 (1.05, 2.80) *

Model 1 is unadjusted. Model 2a is adjusted for age, sex, household income, highest education, ethnicity, smoking status, alcohol consumption, physical activity, myocardial infarction, population density, urbanicity, and province of residence. Model 2b is adjusted for age and sex. * *p* < 0.05.

## Supplementary Materials

The following are available online at https://www.mdpi.com/2072-6643/11/12/3027/s1, Table S1: Diet quality component scores among cancer survivors and non-cancer controls combined, Table S2: Comparison of characteristics between cancer survivors and participants without cancer.
