# Supplementary material for: Diet Quality among Cancer Survivors and Participants without Cancer: A Population-Based, Cross-Sectional Study in the Atlantic Partnership for Tomorrow’s Health Project
Source: Nutrients. 2019 Dec 11;11(12):3027. doi: 10.3390/nu11123027 (PMC6950144; doi:10.3390/nu11123027)
Supplement: Supplementary file 1 [file nutrients-11-03027-s001.pdf]

Supplementary Material

Table S1. Diet quality component scores among cancer survivors and non-cancer controls combined

Component	Mean (SD)
Vegetables and fruit	6.75 (2.78)
Grain products	4.32 (2.38)
Milk and dairy products	6.74 (3.26)
Meat and alternatives	8.95 (2.09)
Snack/dessert/non-diet soft drink	5.63 (3.15)
	**%**
Eat at least one serving of dark green vegetables each day	75.6
Have vegetables and fruit servings more than juice	94.0
Make at least half of grain products whole grain each day	81.5
Avoid oil products while eating bread	10.7
Drink lower fat milk or milk alternatives	85.2
Have meat alternatives such as beans, lentils, and tofu	30.1
Eat fish (at least a serving/day)	38.8
Do not eat saturated fat or its products	78.0
Never or rarely season food with soy sauce or fish sauce at the table	79.8
Never or rarely add salt to food at the table	66.3

Mean diet quality component scores and standard deviations for the first five items of the diet quality scoring scheme (out of 10 with 10 representing greatest adherence) and percentages of participants meeting healthy eating recommendations for the last ten items.

Table S2. Comparison of characteristics between cancer survivors and participants without cancer

	**Cancer survivors** N=14,965	**Participants without cancer** N=5,008
**Age group, N (%)**		
35-39	1440 (9.62)	567 (11.3)
40-44	1698 (11.4)	671 (13.4)
45-49	2199 (14.7)	857 (17.1)
50-54	2692 (18.0)	857 (17.1)
55-59	2788 (18.6)	820 (16.4)
60-64	2534 (16.9)	788 (15.7)
65-69	1614 (10.8)	448 (8.95)
**Sex**		
Female	9705 (64.9)	4092 (81.7)
Male	5260 (35.2)	916 (18.3)
**Marital status**		
Living without partners	2856 (19.1)	960 (19.2)
Living with partners	12077 (80.9)	4038 (80.8)
**Ethnicity**		
White	13006 (93.0)	4412 (93.6)
Non-white	975 (6.97)	304 (6.45)
**Household income**		
0-24,999	679 (4.81)	173 (3.66)
25,000-49,999	2483 (17.6)	730 (15.5)
50,000-74,999	3057 (21.7)	916 (19.4)
75,000-149,999	6109 (43.3)	2193 (46.4)
>150,000	1784 (12.6)	711 (15.1)
**Education (completed)**		
≤ high school	3140 (21.1)	696 (13.9)
college	6182 (41.5)	1913 (38.3)
≥ Bachelor's degree	5585 (37.6)	2390 (47.8)
**Smoking status**[‡]		
Never	7319 (49.3)	2748 (55.3)
Former	5894 (39.7)	1881 (37.9)
Occasional	380 (2.56)	120 (2.42)
Regular	1243 (8.38)	217 (4.37)
**Alcohol consumption**[§]		
Abstainer	646 (4.34)	184 (3.69)
Former drinker	1048 (7.04)	284 (5.69)
Occasional drinker	6134 (41.2)	2145 (43.0)
Regular drinker	4592 (30.9)	1662 (33.3)
Habitual drinker	2456 (16.5)	714 (14.3)
**Physical activity**		
Low	3338 (23.3)	641 (13.3)
Moderate	4482 (31.3)	1394 (28.9)

High	6511 (45.4)	2785 (57.8)
**BMI**		
Normal	3994 (30.9)	2825 (29.5)
Underweight	77 (0.60)	55 (0.57)
Overweight	4922 (38.0)	3725 (38.9)
Obese	3947 (30.5)	2975 (31.1)
**Diabetes**		
Yes	749 (5.06)	249 (5.02)
No	14068 (94.9)	4713 (95.0)
**Myocardial infarction**		
Yes	288 (1.94)	65 (1.31)
No	14577 (98.1)	4912 (98.7)
**Urbanicity**		
Urban	10689 (71.4)	3658 (73.0)
Rural	4276 (28.6)	1350 (27.0)
**Province**		
NL	1993 (13.3)	519 (10.4)
PEI	290 (1.94)	87 (1.74)
NS	9394 (62.8)	3177 (63.4)
NB	3288 (22.0)	1225 (24.5)

Non-smoker: has never smoked, former: has smoked at least 100 cigarettes but not within the past 30 days, occasional: smoked at least once within the past 30 days but not daily, regular: smoked daily. All other participants were categorized as non-smokers. Abstainer: never consumes alcohol, former: has consumed alcohol before but not over the past 12 months, occasional: ≤2–3 drinks month over the past 12 months, regular: drinks ≥ once/week but ≤2–3 times/week, habitual drinkers: drinks ≥ 4–5 times/week. NB; New Brunswick, NL; Newfoundland, NS; Nova Scotia, PEI; Prince Edward Island
